# Influenza A Virus Infection Induces Preferential Increases in Long-Chain Ceramides

**DOI:** 10.3390/v18030339

**Published:** 2026-03-10

**Authors:** Savannah McKenna, Kwang Il Jung, Barbara Sumner, Jennifer J. Wolf, Lloyd W. Sumner, Bumsuk Hahm

**Affiliations:** 1 Department of Surgery, University of Missouri, Columbia, MO 65212, USA; 2 Department of Molecular Microbiology and Immunology, University of Missouri, Columbia, MO 65212, USA; 3 Metabolomics Center, University of Missouri, Columbia, MO 65212, USAsumnerlw@missouri.edu (L.W.S.); 4 Interdisciplinary Plant Group, Christopher S Bond Life Sciences Center, Division of Biochemistry, College of Agriculture, Food and Natural Resources, University of Missouri, Columbia, MO 65212, USA

**Keywords:** influenza virus, ceramides, lipidomics

## Abstract

Influenza is a persistent public health concern worldwide. The elucidation of influenza A virus (IAV)–host interactions and the identification of host factors that regulate IAV infection would be beneficial for combating and treating the disease. Ceramides, comprising a host sphingolipid family, have been shown to regulate virus infections. However, the effect of IAV on individual ceramides remains unknown. This study aimed to investigate the changes in ceramide species during the infection of human lung epithelial A549 cells and human primary tracheal epithelial cells with IAV. We established a method utilizing UHPLC-MS analysis to measure individual ceramides (C14- to C26-ceramide). The results indicate that two main ceramide species, C16- and C24-ceramide, constitute approximately 80% of the ceramide population in human respiratory epithelial cells. Following IAV infection, these ceramides were found to undergo a shift in abundance, with a reduction in C16-ceramide and an increase in C24-ceramide, under various infection conditions. Primarily, IAV infection led to an increase in multiple long-chain ceramides. These findings could provide details for understanding how the ceramide system is disrupted during influenza virus infection and to further support the ongoing efforts to understand influenza–host interactions.

## 1. Introduction

Influenza continues to pose threats to human health, causing yearly epidemics in countries worldwide. Compounded by the limited availability of antiviral therapeutics to combat infections, influenza virus leads to significant morbidity and mortality [1]. Due to the virus nature of undergoing mutations and reassortments, there is a considerable concern that the virus will develop resistance to the currently available antivirals [2]. While both IAV and influenza B virus circulate and cause yearly epidemics, IAV is responsible for over 75% of cases and hosts pandemic potential [3]. As such, it is increasingly important to investigate IAV–host interactions and identify additional therapeutic targets. One strategy for the development of universal influenza antivirals includes targeting host factors, circumventing complications arising from viral mutations.

Ceramides comprise a family of sphingolipids, i.e., bioactive lipid molecules involved in a wide range of cellular and host defense mechanisms [4,5]. Ceramide analogs have been shown to affect IAV replication and promote the activation of dendritic cells upon IAV infection [6,7]. Studies have also shown changes in ceramides during influenza virus infection [7,8,9,10]. Additional elements of the sphingolipid system, including other sphingolipids and sphingolipid-metabolizing enzymes, have been shown to convey both anti- and proviral characteristics during viral infections in vitro and in vivo [11,12,13,14,15,16,17,18,19,20]. Recently, ceramide synthase (CerS) 4, but not CerS1, was reported to interfere with the replication of influenza viruses, suggesting that specific ceramide species produced by differential CerS may have distinct functions during influenza [15]. As such, a deeper understanding of the interactions between the sphingolipid system, including the ceramide network, and viruses would be beneficial.

Ceramides are made up of a sphingoid base, a hydroxyl group, and a fatty acid. The length of the attached fatty acid determines the ceramide species; for example, a ceramide containing a fatty acid with 16 carbons would be designated as C16-ceramide. While studies have shown significant changes in total ceramides following IAV infection [7,8,9,10], there is a lack of studies investigating the changes in individual ceramide species upon infection. In order to better study changes in the ceramide network during influenza virus infection, we sought to investigate the specific fluctuations in individual ceramide species during IAV infection. It was confirmed that there are two main ceramide species in human respiratory epithelial cells, C16- and C24-ceramide, that undergo a shift in abundance during influenza virus infection. Additionally, IAV infection results in an increase in ceramides, particularly in longer-chain ceramides such as C24-ceramide, under various infection conditions.

## 2. Materials and Methods

### 2.1. Cell Culture

Human lung adenocarcinoma epithelial A549 cells (ATCC) were maintained in Dulbecco’s Modified Eagle Medium with high glucose (DMEM) (Gibco) supplemented with 10% fetal bovine serum and 1% of 10,000 U/mL penicillin–streptomycin [11,15,21]. Primary human tracheal epithelial cells (hTECs), which were derived from the excess tissue of human lungs donated for transplant, were provided in a de-identified manner from Dr. Steven Brody (Washington University). The hTECs were maintained in PneumaCult™-NGEx Medium (StemCell Technologies, Vancouver, BC, Canada) supplemented with 2% PneumaCult™-NGEx 50X Supplement (StemCell), 1% hydrocortisone stock solution (96 µg/mL) (StemCell), 0.1% amphotericin B (250 µg/mL), and 1% of 10,000 U/mL penicillin–streptomycin [22]. All cells were kept at 37 °C with 5% CO_2_. Upon collection, cells were washed with PBS, treated with trypsin for approximately 3 min at 37 °C, flushed with DMEM, pelleted at 1000 rpm for 5 min, and resuspended in fresh PBS. Cells were quantified using a Countess 3 (ThermoFisher Scientific, Waltham, MA, USA) automatic cell counter.

### 2.2. Viruses and Infection

Influenza A/Puerto Rico/8/34 (H1N1) (PR8) virus and 2009 pandemic influenza A/CA/04/09 (H1N1) (pH1N1) were amplified in either Madin–Darby Canine Kidney (MDCK) cells or in embryonated chicken eggs as described previously and utilized for cell infections [11,23,24,25]. Cells were infected with viruses at the indicated multiplicity of infection (MOI) in PBS containing 0.3% BSA, 1 mM MgCl_2_, 0.9 mM CaCl_2_, 1% of 10,000 U/mL penicillin–streptomycin, and 1% GlutaMAX™ (100X) (Gibco, Waltham, MA, USA) for 1 h at 37 °C and 5% CO_2_. After incubation, virus-containing PBS was removed and replaced with DMEM supplemented with 0.3% BSA, 1 µg/mL TPCK-treated trypsin (Sigma, St. Louis, MO, USA), 1% of 10,000 U/mL penicillin–streptomycin, and 1% GlutaMAX™ (100X) (Gibco) and returned to the incubator for the indicated duration of time.

### 2.3. Ceramide Extraction

The ceramide extraction protocol was adapted from a previous report [26]. Briefly, after cell collection and quantification, an equal number of cells were transferred to a 4 mL borosilicate glass vial and centrifuged at 1000 rpm for 5 min. Supernatant was removed and cells were resuspended in 0.5 mL LC/MS-grade methanol (OmniSolv^®^, Sigma) and 0.25 mL LC/MS-grade chloroform (OmniSolv^®^, Sigma). A total of 50 µL of Cer/Sph Mixture I internal standard (2.5 µM) (Avanti Polar Lipids, Alabaster, AL, USA) was added to each sample. For experiments including absolute quantification, 50 µL of deuterium-labeled C16-ceramide-d7 (d18:1–d7/16:0) and C24-ceramide-d7 (d18:1–d7/24:0) mixed 1:1 (1.5 µg/mL) was also added to each sample. Samples were vortexed and sonicated for 1 min at room temperature, then incubated overnight in a water bath at 48 °C. Samples were removed from the water bath and cooled to room temperature. A 75 µL volume of 1 M KOH solution in methanol (OmniSolv^®^, Sigma) was added to each sample. Samples were again sonicated for 1 min at room temperature, then incubated at 37 °C for 2 h, shaking at 225 rpm. Samples were cooled to room temperature, and pH was adjusted to neutral by the addition of approximately 3 to 6 µL of glacial acetic acid and vortexed. Using a tabletop centrifuge, samples were spun at 3600× *g* for 30 min for the first extraction. Supernatants were collected and transferred to 2 mL screw top vials (SureSTART™, ThermoFisher). Samples were resuspended in 0.5 mL methanol and 0.25 mL chloroform, vortexed, and centrifuged again at 3600× *g* for 30 min for a second extraction. The supernatant was collected and combined with the prior extraction supernatant. The extracted samples were dried completely using a nitrogen evaporator. Dried residue was resuspended in 100 µL methanol to be used for ceramide measurement.

### 2.4. Ceramide Detection

Ceramide extract samples were centrifuged at 3500× *g* briefly to remove any remaining insoluble residue. The supernatant was then transferred to an autosampler vial with 150 µL limited volume insert for UHPLC-MS/MS (ultra-high-pressure liquid chromatography–mass spectrometry/mass spectrometry) analysis.

Samples were injected (2 µL injection volume), separated, and mass analyzed using a Waters Acquity UHPLC (Waters Corporation, Milford, MA, USA) coupled with a Waters Xevo TQ Absolute triple quadrupole mass spectrometer (Waters Corporation). Ceramides were separated using gradient elution with two buffered mobile phases (2 mM ammonium formate in water = A, 2 mM ammonium formate in methanol = B) and a Waters Acquity C18 BEH column (Waters Corporation) (2.1 × 150 mm, 1.7 µm thickness). A 20 min gradient was used with a flow rate of 0.30 mL/min and a column temperature of 60 °C. The gradient was initially 50% A and 50% B, but increased to 80% B at 2.30 min, then 100% B at 10.30 min and held until 17.30 min. At this time, the gradient returned to 50% B and was allowed to equilibrate for 3.0 min.

The Waters MassLynx software (V4.2) was used to acquire and process the UHPLC-MS/MS data. The Intellistart software within MassLynx (V4.2) was used to optimize the transitions for the Multiple Reaction Monitoring (MRM) of the sphingolipids and ceramides. Data was acquired using the +ESI (electrospray ionization) mode. In addition to the quantifier transition, a qualifier transition was included to confirm the compound assignment. C16- and C24-ceramides were quantified using deuterated internal standards, C16-d7 and C24-d7 (Avanti Polar Lipids). A calibration curve for each was generated using a dilution series ranging from 0.025 µg/mL to 2.5 µg/mL. Other species were normalized by using internal standards from the Avanti Polar Lipids Sph/Cer I mix to determine relative measurements. The MRM quantifier transitions, optimized cone voltages, and collision energies for the measured compounds and internal standards are shown in Table 1. The Waters TargetLynx (V4.1.1.0) software package was used to process the data and quantify the compounds.

### 2.5. Western Blot Analysis

Western blot was conducted as described previously [14,27,28]. Briefly, denatured polypeptides from cell lysates were resolved by SDS-PAGE and transferred onto nitrocellulose membranes (Bio-Rad Laboratories, Hercules, CA, USA). After overnight blocking and subsequent overnight incubation with a primary antibody at 4 °C, membrane-bound primary antibodies were detected using HRP-conjugated secondary antibodies. The signals were visualized using an Odyssey Fc imaging system (Li-Cor), developed for 30 s with chemiluminescence and 700 channels, and processed with the Image Studio software (Li-Cor, v5.2). Anti-GAPDH antibody was purchased from Cell Signaling Technology (Danvers, MA, USA) (5174S). Antibodies against IAV NP and M1 were purchased from Abcam (Cambridge, MA, USA) (ab43821 and ab22396, respectively). Anti-rabbit IgG HRP and anti-mouse IgG HRP were purchased from Cell Signaling Technology (7074S and 7076S, respectively).

### 2.6. Statistical Analysis

Ceramide relative measurements were determined for each sample as an instrument response. Response values were calculated as the area detected for the individual ceramide species (Cer) for the sample divided by the area detected for the corresponding internal standard (IS) for the sample ($Sample\;response=\;\frac{{Area}_{Cer}}{{Area}_{IS}}$). Unnaturally occurring C12-ceramide was used as an internal standard for C14-C18-ceramide, while unnaturally occurring C25-ceramide was used as an internal standard for C20-C26-ceramide. Although the internal standards were added in equal amounts, they were detected at unequal extents. In order to correct for the unequal detection of internal standards, which would unequally skew the ceramide measurements, we multiplied C20- to C26-ceramide sample response by the quotient of the average area of C25-ceramide/C12-ceramide that were used as internal standards (${Sample\;response}_{C20-C26}\times \;\frac{{Avg\;Area}_{C25\;IS}}{{Avg\;Area}_{C12\;IS}}$). The absolute quantification of C16- and C24-ceramide was performed using a standard curve generated for a concentration series of deuterium-labeled standards (concentrations ranging from 0.025 to 2.5 µg/mL). The relative abundance of ceramides was calculated using instrument response values for each ceramide species divided by total response per sample, averaged among each experimental group ($Abundance=\;{Avg}_{Group}(\frac{{Sample\;response}_{Cer}}{{Sample\;response}_{Total}})$). Statistical significance was determined via Student’s *t*-test or one-way ANOVA with Tukey’s HSD post hoc multiple comparison correction using GraphPad Prism (V10.2.0) (*p* < 0.05). Error bars on all graphs represent standard deviation.

## 3. Results

### 3.1. IAV Infection Alters Cellular Ceramides, with a Preferential Increase in Long-Chain Ceramides

**Figure 1:**
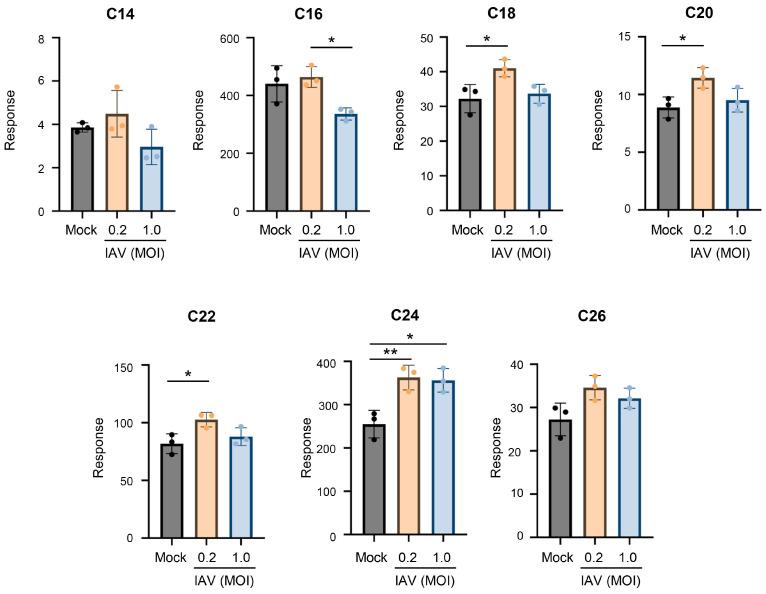
Ceramides tend to increase during influenza virus infection, with a preferential increase in long-chain ceramides. A549 cells were either mock-infected or infected with IAV (pH1N1) at an MOI of 0.2 or MOI of 1.0 for 24 h. Relative amounts of C14-ceramide, C16-ceramide, C18-ceramide, C20-ceramide, C22-ceramide, C24-ceramide, and C26-ceramide were detected via liquid chromatography–mass spectrometry. Response values were normalized using an internal standard mixture. The experiment was run with 3 biological replicates per group. Data is representative of 3 independent experiments, each experiment with *n* = 3–4/group. Significance was determined by one-way ANOVA. * <0.05, ** <0.01.

To investigate if IAV induces any changes in individual ceramide species, we have established an assay system for ceramide measurements using UHPLC-MS/MS and MRM as described in the Materials and Methods section. Ceramide mixture internal standard containing unnatural ceramides, i.e., C12- and C25-ceramide, was added to all samples before the extraction of the lipids to compensate for any possible changes made through the extraction and measurement processes. In this study, human lung epithelial A549 cells were mock-infected or infected with pandemic influenza A/CA/04/09 (H1N1) (pH1N1) at an MOI of 0.2 or an MOI of 1.0 for 24 h. The relative amounts of individual ceramide species (C14- to C26-ceramides) were measured from cellular lipid extracts to determine changes in the ceramides in infected cells compared to mock-infected cells (Figure 1). IAV infection at the lower MOI of 0.2 significantly increased C18-, C20-, C22-, and C24-ceramide compared to mock infection. However, the shorter-chain ceramides, i.e., C14- and C16-ceramide, showed no significant change with infection. IAV infection at an MOI of 1.0 also induced a preferential increase in longer-chain ceramides. This is particularly apparent when considering the long-chain C24-ceramide. These results indicate that IAV infection caused increases in longer-chain ceramide species in human lung epithelial A549 cells.

### 3.2. Human Lung Epithelial A549 Cells Have Two Main Ceramide Species, C16- and C24-Ceramide, That Undergo a Shift During IAV Infection

**Figure 2:**
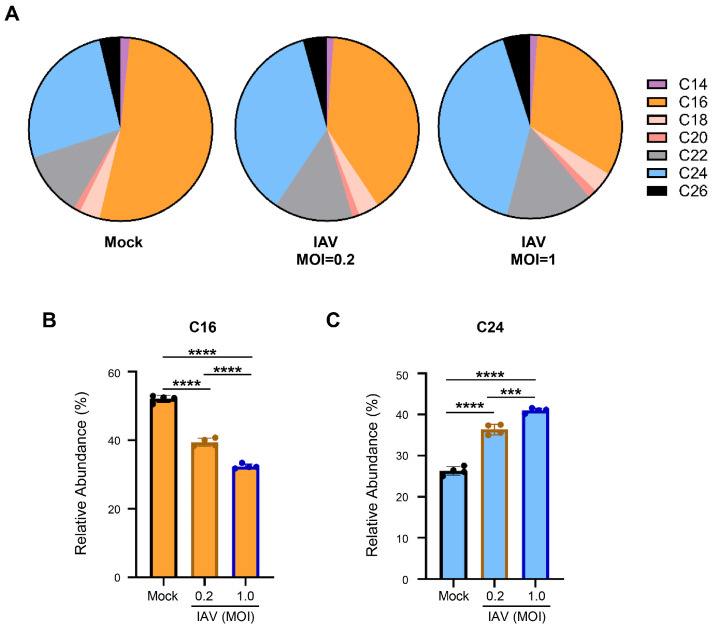
IAV infection skews cellular ceramide abundance away from C16-ceramide and toward C24-ceramide. A549 cells were either mock-infected or infected with IAV (pH1N1) at an MOI of 0.2 or an MOI of 1.0 for 24 h. Relative amounts of individual ceramide species were detected via liquid chromatography–mass spectrometry. Relative abundance was calculated as the proportion of the detected response corresponding to each individual ceramide species out of the total response (**A**). The relative abundance for the two major ceramide species, C16-ceramide (**B**) and C24-ceramide (**C**), are shown individually. The experiment was run with 4 biological replicates per group. Data is representative of 3 independent experiments, each experiment with *n* = 3–4/group. Significance was determined by one-way ANOVA. *** <0.001, **** <0.0001.

Our analysis of ceramide composition based on the measurements demonstrated the prevalence of two main ceramide species within human lung epithelial A549 cells—C16-ceramide and C24-ceramide (Figure 2). This is in agreement with previous reports from mouse lungs and other human lung cells [29]. Indeed, these two ceramide species account for nearly 80% of total ceramide abundance (Figure 2A). Interestingly, we consistently observed a significant shift during IAV infection where the abundance of C16-ceramide decreased (mock: 52%, IAV: 39% at an MOI of 0.2 and 32% at an MOI of 1.0), whereas the abundance of C24-ceramide increased (mock: 26%, IAV: 36% at an MOI of 0.2 and 41% at an MOI of 1.0) (Figure 2B,C). The reduction in the abundance of C16-ceramide and the increase in the abundance of C24-ceramide were more severe during infections conducted at the higher MOI of 1.0.

### 3.3. IAV Infection Under Various Conditions Results in Consistent Changes in Cellular Ceramides with a Preferred Increase in Long-Chain Ceramides

**Figure 3:**
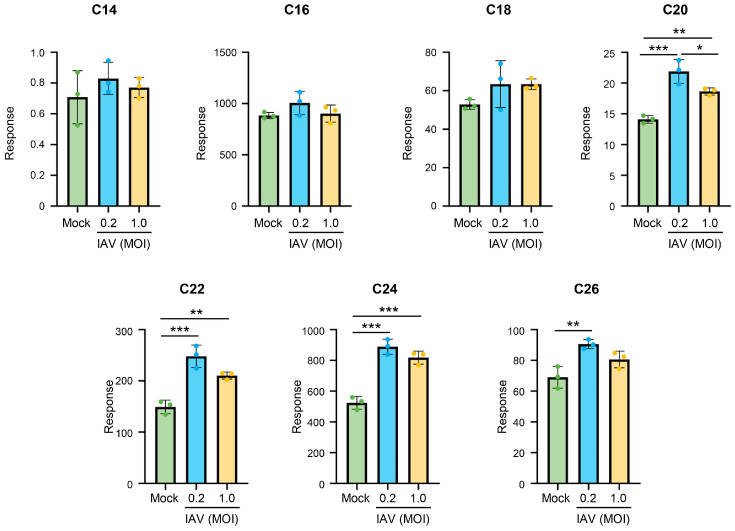
Infection with IAV PR8 strain retains a trend of increasing ceramides during infection, with a preferential increase in long-chain ceramides. A549 cells were either mock-infected or infected with IAV (PR8) at an MOI of 0.2 or an MOI of 1.0 for 24 h. Relative amounts of C14-ceramide, C16-ceramide, C18-ceramide, C20-ceramide, C22-ceramide, C24-ceramide, and C26-ceramide were detected via liquid chromatography–mass spectrometry. Response values were normalized using an internal standard mixture. The experiment was run with 3 biological replicates per group. Significance was determined by one-way ANOVA. * <0.05, ** <0.01, *** <0.001.

To determine whether IAV-induced changes in ceramide metabolism were specific to the IAV strain (pH1N1) being used, we conducted additional experiments utilizing influenza A/Puerto Rico/8/34 (H1N1) (PR8) under the same conditions: A549 cells were mock-infected or infected with PR8 at an MOI of 0.2 or an MOI of 1.0 for 24 h, followed by ceramide extraction and assessment (Figure 3). PR8 infection at an MOI of 0.2 led to a significant increase in long-chain ceramide species, such as C20-, C22-, C24- and C26-ceramide. Additionally, there was a preferential increase in longer-chain ceramides when cells were infected with the PR8 strain at an MOI of 1.0. The findings suggest that both pH1N1 and PR8 influenza strains induce similar alterations in ceramide species upon infection.

**Figure 4:**
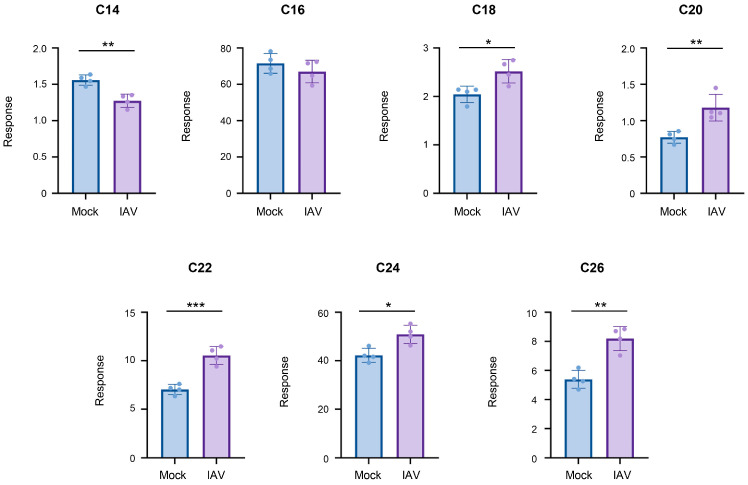
Ceramides tend to increase during a single-cycle of influenza virus infection, with the exception of C14- and C16-ceramide. A549 cells were either mock-infected or infected with IAV (pH1N1) at an MOI of 5.0 for 6 h. Relative amounts of C14-ceramide, C16-ceramide, C18-ceramide, C20-ceramide, C22-ceramide, C24-ceramide, and C26-ceramide were detected via liquid chromatography–mass spectrometry. Response values were normalized using an internal standard mixture. The experiment was run with 4 biological replicates per group. Data is representative of 2 independent experiments, each experiment with *n* = 4/group. Significance was determined by Student’s *t*-test. * <0.05, ** <0.01, *** <0.001.

To mitigate changes in ceramide populations that may be due to the effects of uninfected cells, we conducted infections with pH1N1 virus at a high MOI of 5.0 and limited infection to 6 h (Figure 4). Halting infection and collecting cells at the early time point should limit the infection to a single-cycle of replication, while infection at an MOI of 5.0 should ensure a majority of cells become infected, allowing for the constrained evaluation of ceramide population changes specifically in IAV-infected cells. Under these conditions, the longer-chain ceramides, specifically C18-, C20-, C22-, C24-, and C26-ceramide, significantly increased. However, C14-ceramide significantly decreased, while C16-ceramide was unchanged by infection.

**Figure 5:**
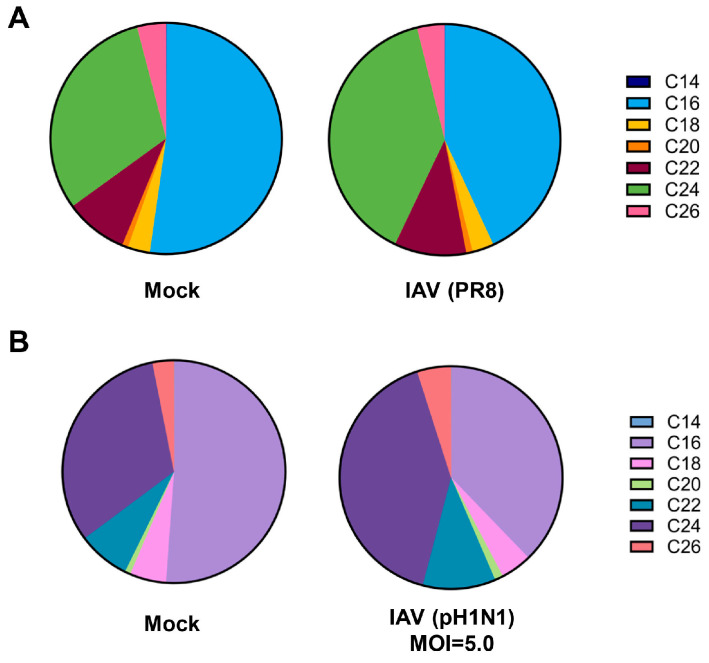
Infection with IAV PR8 strain and single-cycle infection show a similar shift in ceramide abundance away from C16-ceramide toward C24-ceramide. A549 cells were either mock-infected or infected with IAV (PR8) at an MOI of 1.0 for 24 h (**A**) or infected with IAV (pH1N1) at an MOI of 5.0 for 6 h (**B**). Relative amounts of individual ceramide species were detected via liquid chromatography–mass spectrometry. Relative abundance was calculated as the proportion of the detected response corresponding to each individual ceramide species out of the total response.

In both conditions, PR8 infected at an MOI of 1.0 and pH1N1 infected at an MOI of 5.0, C16-ceramide and C24-ceramide remained the main ceramide species (Figure 5). Importantly, the previously noted shift in C16- and C24-ceramide abundance was recapitulated under these infection conditions. Specifically, PR8 virus infection (Figure 5A) as well as a short-term infection with pH1N1 at an MOI of 5.0 (Figure 5B) led to a reduction in C16-ceramide and an increase in C24-ceramide abundance.

**Figure 6:**
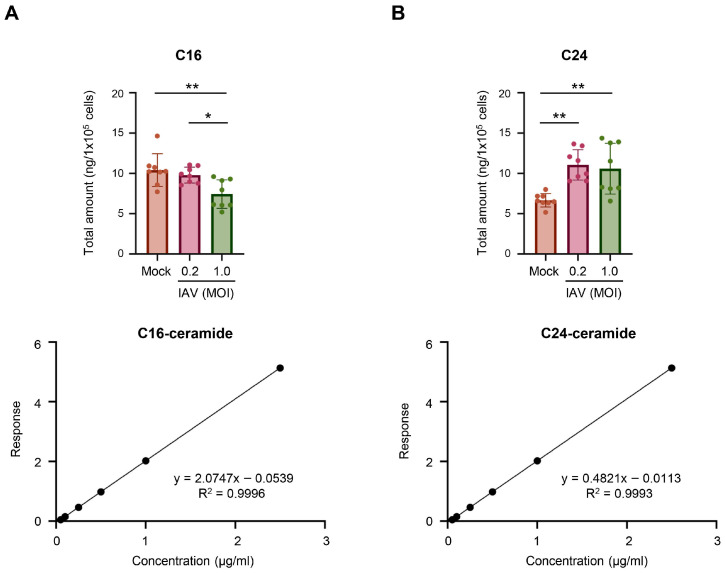
Measurements of absolute amounts of C16-ceramide and C24-ceramide during IAV infection. A549 cells were either mock-infected or infected with IAV (pH1N1) at an MOI of 0.2 or an MOI of 1.0 for 24 h. Absolute amounts of C16-ceramide (**A**) and C24-ceramide (**B**) were measured from 100,000 cells in a volume of 100 µL via liquid chromatography–mass spectrometry. Deuterium-labeled C16-ceramide (**A**) and C24-ceramide (**B**) were included as internal controls to generate the corresponding standard curve as shown in the bottom panels (for C16-ceramide, R^2^ = 0.9996; for C24-ceramide, R^2^ = 0.9993; detection range = 0.025 − 2.5 µg/mL). The data were pooled from 2 independent experiments, where each experiment contained 4 biological replicates per group. Significance was determined by one-way ANOVA. * <0.05, ** <0.01.

As C16-ceramide and C24-ceramide were consistently shown to be the two major ceramide species in A549 cells, these ceramides were more specifically measured for their quantities altered during IAV infection. Utilizing deuterium-labeled internal standards, we were able to absolutely quantify the amount of C16-ceramide and C24-ceramide in the samples (Figure 6). After A549 cells were infected with pH1N1 virus at an MOI of 0.2 or 1.0 for 24 h, C16-ceramide and C24-ceramide were measured. The total amount of C16-ceramide significantly decreased in cells infected at an MOI of 1.0, but not at an MOI of 0.2 (Figure 6A). As anticipated, the total amount of C24-ceramide significantly increased under both infection conditions (Figure 6B).

### 3.4. IAV Infection Alters Cellular Ceramides in Human Primary Tracheal Epithelial Cells, Resulting in Greater Increase in Long-Chain Ceramides

**Figure 7:**
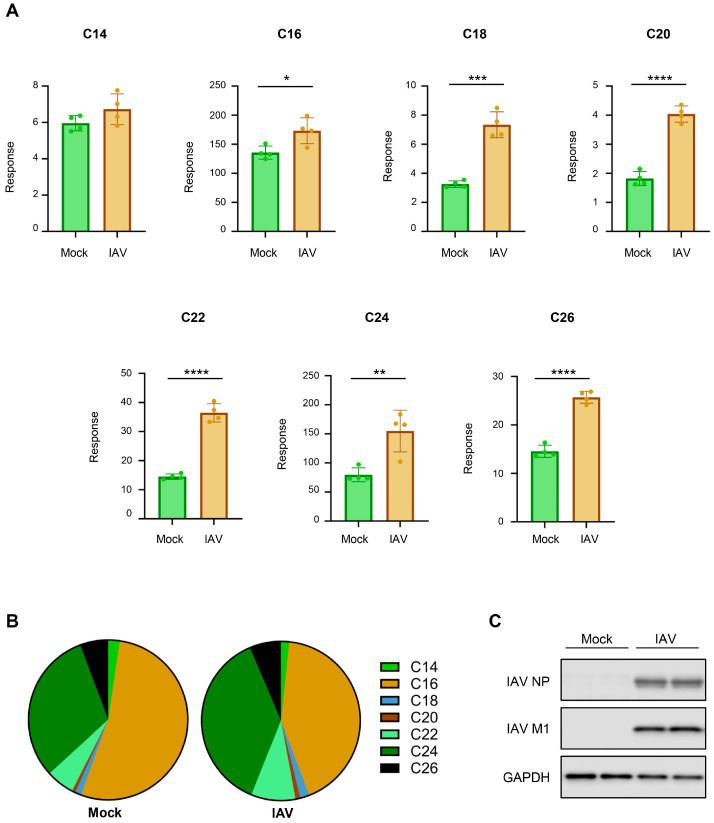
Infection of human primary tracheal epithelial cells (hTECs) with IAV increases cellular ceramides. Human primary tracheal epithelial cells were either mock-infected or infected with IAV (pH1N1) at an MOI of 0.2 for 24 h. Relative amounts of C14-ceramide, C16-ceramide, C18-ceramide, C20-ceramide, C22-ceramide, C24-ceramide, and C26-ceramide (**A**) were detected via liquid chromatography–mass spectrometry. Response values were normalized using an internal standard mixture. Relative abundance was calculated as the proportion of the detected response corresponding to each individual ceramide species out of the total response (**B**). hTECs were either mock-infected or infected with IAV (pH1N1) at an MOI of 0.2 for 24 h. Cellular lysates were utilized for Western blot to detect the levels of viral NP, viral M1, and GAPDH (**C**). Ceramide measurements were conducted with 4 biological replicates per group. Data is representative of two independent experiments, each experiment with *n* = 4/group. Significance was determined by Student’s *t*-test. * <0.05, ** <0.01, *** <0.001, **** <0.0001.

Next, we determined if the findings from the A549 cell line could be extended to more physiologically relevant cells, such as human primary epithelial cells. For this purpose, the experiment shown in Figure 1 was repeated using human primary tracheal epithelial cells (hTECs), which were derived from the excess tissue of human lungs donated for transplantation [11,22]. Due to these cells seeming more susceptible to IAV infection in our experimental condition, the hTECs were mock-infected or infected with pH1N1 virus at an MOI of 0.2 for 24 h, followed by ceramide extraction and measurement (Figure 7).

When primary hTECs were infected with IAV, there was an increase across many ceramide species, with notable changes in C18-, C20-, C22-, C24-, and C26-ceramide (Figure 7A). However, IAV infection did not significantly change C14-ceramide in hTECs. When the relative composition of the individual ceramide species was calculated, C16- and C24-ceramide were found to be two major ceramides in hTECs and constitute 80% of the ceramide population (Figure 7B). More importantly, we still observed an overall reduction in the abundance of C16-ceramide (mock: 50%, IAV: 39%) and an increase in the abundance of C24-ceramide (mock: 30%, IAV: 39%), agreeing with previous results from experiments conducted in A549 cells. IAV infection of hTECs in the experimental condition was confirmed using Western blot analysis (Figure 7C).

## 4. Discussion

Utilizing an in-house established UHPLC-MS/MS analysis of ceramides, this study examined changes in individual ceramide species during IAV infection of human lung epithelial A549 cells and human primary tracheal epithelial cells. Upon infection, long-chain ceramides were preferentially increased. Two ceramide species, C16- and C24-ceramide, were identified as predominant ceramide species and shown to undergo a shift during infection, with a reduction in C16-ceramide and an increase in C24-ceramide.

Previous studies have suggested that total ceramides generally increase during IAV infection [8,9,10]. However, these studies did not differentiate between different ceramide species. Our findings suggest that ceramides are dysregulated during influenza virus infection in both conditions of single-cycle replication as well as multiple cycles of replication. When A549 cells were infected with IAV at a low MOI (MOI of 0.2), we observed a tendency for more increases in several ceramide species when compared to those from cells infected at a higher MOI (MOI of 1.0) (Figure 1). This implies a possible role of uninfected cells in increasing ceramides. Further, the treatment of cells with inflammatory cytokines, such as IL-1 and TNF-α, has been reported to stimulate ceramide accumulation [30,31]. Under lower infection conditions, the cytokines produced from virus-infected cells may affect uninfected surrounding cells differently from virus-infected cells. To reduce the potential contribution of uninfected cells on ceramide population changes, a higher MOI of 5.0 was used. However, during this condition the majority of ceramides still increased, suggesting that long-chain ceramides can increase in virus-infected cells without abundant uninfected cells.

The preferential increase in long-chain ceramides may be explained in a few different ways. Ceramides are synthesized by a family of enzymes known as ceramide synthase (CerS), consisting of six different enzymes (CerS1-6). Each of these synthases has specificity for ceramides of a specific length [32,33,34,35,36]. It is possible that, upon influenza virus infection, there is enhanced activation of the ceramide synthases corresponding to synthesis of long-chain ceramides, promoting a greater production of long-chain ceramides, such as C24-ceramide. A recent study has reported that CerS4 reduced IAV replication through the inhibition of JNK [15]. CerS4 preferentially synthesizes C18- and C20-ceramide. However, in the present study, both C18- and C20-ceramide were found to be minor ceramide species, contributing to approximately 3–5% of ceramide abundance collectively. The major ceramide species, C16- and C24-ceramide, which constitute approximately 80% of the ceramide population collectively, are preferentially synthesized by CerS5/CerS6 and CerS2/CerS3, respectively. Whether these enzymes are responsible for the shift in C16-ceramide to C24-ceramide remains to be investigated in the context of IAV infection. In the opposite direction, ceramide can be broken down to produce sphingosine by another type of ceramide-metabolizing enzyme, ceramidase. Like ceramide synthases, multiple different ceramidases (acid, neutral, and alkaline ceramidases) have specificity for ceramides of varying lengths [37,38,39]. Thus, it is possible that, during influenza virus infection, there is a specific activation of ceramidase that results in a greater accumulation of long-chain ceramides. Furthermore, ceramidases can also be regulated in part by CerS and subsequent ceramide accumulation, as shown when CerS6 led to C16-ceramide accumulation and acid ceramidase activation [40]. Acid ceramidase, which preferentially cleaves shorter-chain ceramides, has been shown to dysregulate type I interferon and CD8+ T cell responses during viral infection [41,42,43]. Ceramides can also be recycled from more complex sphingolipids within the cell or produced by sphingomyelin hydrolysis. This highlights the complexity of the sphingolipid system and the many avenues that may be contributing to the observed changes in the different ceramide species during influenza virus infection. Thus, comprehensive investigation is needed in order to determine if the shift contributes to robust virus replication or host defense to infection.

It is interesting to consider that ceramide has been shown to regulate a variety of host defense mechanisms, such as activating immune cells, modulating inflammatory responses, and managing cell apoptosis [6,42,43,44,45,46]. Previous studies indicate that exogenously supplied ceramide analogs regulate influenza virus replication in vitro [7]. Since the ceramide analog can increase the total level of ceramide in cells, it suggests that the increased ceramides could impair influenza virus replication. However, CerS4, but not CerS1, inhibits the replication of influenza viruses, indicating that ceramide species may have different functions during infection [15]. These findings further emphasize the importance of future investigation of individual ceramides and respective CerS functions during virus infection.

Our study begins investigating the changes in individual ceramides during IAV infection, providing a basis for future investigations aiming to better explain the interactions between ceramides and influenza virus. This knowledge will contribute to understanding the interplay between the virus and host sphingolipids and provide key information in identifying how sphingolipid metabolism could be targeted for antiviral therapeutic potential.

## Figures and Tables

**Table 1:** Details of Multiple Reaction Monitoring.

Compound	Quantifying Transition	Cone Voltage	Collision Energy
C12 *	482.50 → 81.80	20 V	48 eV
C14	510.50 → 264.25	18 V	24 eV
C16	538.50 → 264.25	26 V	26 eV
C16-d7 *	545.50 → 271.25	16 V	26 eV
C18	566.50 → 264.25	20 V	26 eV
C20	594.57 → 264.25	8 V	34 eV
C22	622.60 → 264.25	8 V	30 eV
C24	650.64 → 264.25	20 V	32 eV
C24-d7 *	657.67 → 271.25	10 V	28 eV
C25 *	664.65 → 264.25	28 V	34 eV
C26	678.67 → 264.25	32 V	30 eV

* Compounds (which are not naturally occurring) were used as internal standards. The C12-ceramide was used as an internal standard for C14- to C18-ceramide; the C25-ceramide was used as an internal standard for C20-, C22-, and C26-ceramide.

## Data Availability

The raw data supporting the conclusions of this article will be made available by the authors on request.
